# Sialic Acid Glycobiology Unveils *Trypanosoma cruzi* Trypomastigote Membrane Physiology

**DOI:** 10.1371/journal.ppat.1005559

**Published:** 2016-04-08

**Authors:** Andrés B. Lantos, Giannina Carlevaro, Beatriz Araoz, Pablo Ruiz Diaz, María de los Milagros Camara, Carlos A. Buscaglia, Mariano Bossi, Hai Yu, Xi Chen, Carolyn R. Bertozzi, Juan Mucci, Oscar Campetella

**Affiliations:** 1 Instituto de Investigaciones Biotecnológicas, Universidad Nacional de San Martín, San Martín, Buenos Aires, Argentina and Consejo Nacional de Investigaciones Científicas y Técnicas, Buenos Aires, Argentina; 2 Laboratorio de Nanoscopías Fotónicas, INQUIMAE, Facultad de Ciencias Exactas y Naturales, Universidad de Buenos Aires, Buenos Aires, Argentina; 3 Department of Chemistry, University of California, Davis, Davis, California, United States of America; 4 Department of Chemistry, Stanford University and Howard Hughes Medical Institute, Stanford, California, United States of America; University of Melbourne, AUSTRALIA

## Abstract

*Trypanosoma cruzi*, the flagellate protozoan agent of Chagas disease or American trypanosomiasis, is unable to synthesize sialic acids *de novo*. Mucins and *trans*-sialidase (TS) are substrate and enzyme, respectively, of the glycobiological system that scavenges sialic acid from the host in a crucial interplay for *T*. *cruzi* life cycle. The acquisition of the sialyl residue allows the parasite to avoid lysis by serum factors and to interact with the host cell. A major drawback to studying the sialylation kinetics and turnover of the trypomastigote glycoconjugates is the difficulty to identify and follow the recently acquired sialyl residues. To tackle this issue, we followed an unnatural sugar approach as bioorthogonal chemical reporters, where the use of azidosialyl residues allowed identifying the acquired sugar. Advanced microscopy techniques, together with biochemical methods, were used to study the trypomastigote membrane from its glycobiological perspective. Main sialyl acceptors were identified as mucins by biochemical procedures and protein markers. Together with determining their shedding and turnover rates, we also report that several membrane proteins, including TS and its substrates, both glycosylphosphatidylinositol-anchored proteins, are separately distributed on parasite surface and contained in different and highly stable membrane microdomains. Notably, labeling for α(1,3)Galactosyl residues only partially colocalize with sialylated mucins, indicating that two species of glycosylated mucins do exist, which are segregated at the parasite surface. Moreover, sialylated mucins were included in lipid-raft-domains, whereas TS molecules are not. The location of the surface-anchored TS resulted too far off as to be capable to sialylate mucins, a role played by the shed TS instead. Phosphatidylinositol-phospholipase-C activity is actually not present in trypomastigotes. Therefore, shedding of TS occurs via microvesicles instead of as a fully soluble form.

## Author Summary

*Trypanosoma cruzi* is a flagellated protozoan parasite whose life cycle includes stages alternating between insect and mammal hosts. The membrane of the infective trypomastigote is equipped to allow survival in blood and to invade cells where it proliferates. Here we provide a novel description of the localization in separate microdomains of the *trans*-sialidase and mucins, enzyme and substrate, respectively, that play the main role in the crucial scavenging of sialic acids from the host, which allows parasite survival and infection. Even though these proteins, key in the parasite biology, share similar anchoring to the membrane, their puzzling segregation evidences the complexity of the trypanosome virulence mechanisms and protein trafficking. Instead of as soluble proteins, they are shed in microvesicles, a finding relevant to their known role in the pathogenesis of *T*. *cruzi* infection. This work provides an integrated view that explores these phenomena at the nanoscale and allows to build a model for the trypomastigote membrane physiology.

## Introduction

*Trypanosoma cruzi*, the agent of Chagas disease or American trypanosomiasis, is unable to synthesize sialic acids, entirely depending on a modified α(2,3)-sialidase known as *trans-*sialidase (TS) to scavenge them from the host’s glycoconjugates through a glycosyl-transfer reaction. TS constitute a family of glycosylphosphatidylinositol (GPI)-anchored proteins consisting of a catalytic domain followed by an extension of a repetitive motif composed by twelve aminoacid residues known as SAPA. TS awakens great interest and has been extensively studied based on its biochemical properties, relevance in parasite cell biology and in the induction of strong alterations on the host immune response [1–3]. GPI-anchored-TS is expressed in the infective parasite (trypomastigote) stage and can be easily found in sera from acutely infected mammals or in trypomastigote-conditioned medium after shedding [4, 5]. Hence, TS is considered a virulence factor of *T*. *cruzi*.

In the presence of suitable donors (*i*.*e*. glycans with an α(2–3)-sialyl residue linked to a terminal β-Galactose), the parasite’s surface becomes rapidly sialylated by TS [6]. Sialyl acceptor molecules and TS constitute a glycobiological system crucial in the *T*. *cruzi* life cycle [7]. Sialylation of the parasite surface by TS is a requisite to avoid lysis by serum factors [8, 9] and thus sialylation occurs as acceptor glycoconjugates become exposed on the surface. Parasite glycoconjugates conform a dense negatively charged coat [8], which is also associated to the invasion of the host cell [7]. Although probably not the unique acceptors available for the TS-transferred sialyl residue, mucins are considered as its main targets [8, 10]. *T*. *cruzi* mucins are a heterogeneous family of heavily *O*-glycosylated molecules (for a review see [11, 12]) that are also shed to the milieu and have strong pro-inflammatory properties on the host [13–15]. Previous genetic, biochemical and proteomic studies revealed that the highly polymorphic *TcMUC* gene family, and particularly those genes belonging to the *TcMUC II* subgroup, code for the peptide scaffolds of 60–200 kDa mucins restricted to the trypomastigote surface and known as tGPI-mucins [16, 17]. Despite their complexity and variations in amino acid sequence, TcMUC II deduced products share a common structure made up of a highly conserved *N*-terminal signal peptide, a variable and central region showing highly biased amino acid composition, in which Thr, Ser, Pro, Gly, and Ala residues together might add up to 60–80% of the total count, and a *C*-terminal GPI attachment signal. Besides the *TcMUC* genes, other families of glycosylated proteins families such as TcTASV [18] and MASP [7] are being discovered. To date, the structure of the *O*-linked oligosaccharides has only been determined for the mucins from the non-infective insect epimastigote stage (known as Gp35/50 mucins) of different strains of *T*. *cruzi* [12]. Little is known about the structure of the *O-*glycans of the trypomastigotes mucins. The larger structures are branched, contain a variable number of Gal*p* units and can be sialylated indicating the presence of ßGal terminal residues [19, 20]. The simplest structures found correspond to Galα(1,3)Galβ(1,4)GlcNAcα epitopes (known as αGal), which are only found in trypomastigote mucins and constitute a main target of protective and lytic antibody responses in Chagasic patients [20, 21].

Due to its biologic relevance in the host/parasite interaction, it is of high interest to fully understand the sialic acid acquisition process together with the distribution of the acceptor molecules on the *T*. *cruzi* surface. A major drawback to analyze the kinetics of the infective trypomastigote acceptors is the difficulty to identify and follow the recently acquired sialyl residues. To tackle this issue, we followed an unnatural sugar approach as bioorthogonal chemical reporters [22, 23]. The use of modified azidosialyl residues allowed us to identify the TS-catalyzed modification of the glycosylation pattern of parasites and cells from the immune system [6] with unnoticeable chemical perturbation. In this way, TS target proteins can be studied to the molecular level with higher selectivity and minimal background in contrast to the use of mucin-directed antibodies or lectins.

In *T*. *cruzi*, it has been proposed that proteins are located in different regions of the parasite surface following a spatial distribution that may be associated with their functions [24]. Recently, we reported that GPI-anchored proteins reach the parasite surface by an unconventional pathway that involves the contractile vacuole [25]. However, even when TS and mucin-like TSSA [26] are GPI-anchored proteins they are trafficked via different routes thus suggesting a strategy to differentially locate them into defined/specific regions of the membrane [25].

To clarify the *T*. *cruzi* trypomastigote membrane biology and structure as well as protein turnover we addressed the TS and sialyl acceptors surface distribution, turnover kinetics, sialylation and shedding processes. Here we report for the first time that several membrane proteins including TS and its targets are separately distributed on *T*. *cruzi* surface and contained in different and highly stable membrane microdomains. This location resulted too far off for the surface-anchored TS to sialylate mucins, a role played by the shed TS instead. In addition, we report that TS is shed only associated to microvesicles and not in a fully soluble form, thus challenging the general assumption that, in trypomastigotes, GPI-anchored proteins are shed after phosphatidylinositol-phospholipase-C (PI-PLC) hydrolysis. We also provide new evidence that mucins are actually the main sialyl acceptor molecules and that at least two glycosylated species of mucins exists based on the presence of sialyl and α(1,3)Galactosyl (αGal) residues.

## Results

### Characterization, shedding and turnover of trypomastigote’s sialyl acceptors

**Fig 1:**
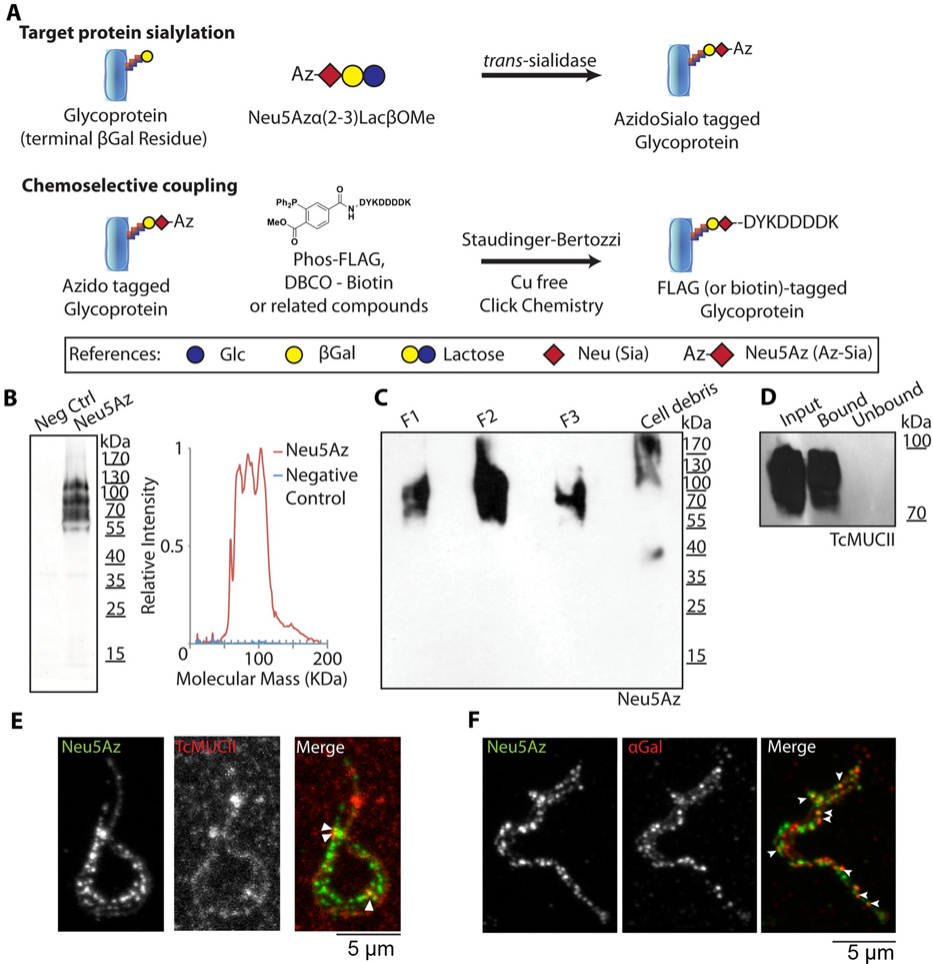
Mucin sialylation and characterization. A) Mucins (or any glycoconjugate bearing a terminal β-galactose) may be tagged by the TS using a Neu5Az donor such as Neu5Azα(2–3)LacβOMe. Then the Azide group may be coupled via the Staudinger-Bertozzi chemistry or the Cu^2+^-free click chemistry to obtain a FLAG or biotin tag ready for detection. B) Western blot of Neu5Az *trans*-sialylated trypomastigote lysates revealing the relative molecular mass distribution of acceptor molecules. A line profile of the blot is also plotted. Neg Ctrl: Negative control. C) Neu5Az *trans*-sialylated parasites were submitted to organic solvents extraction as described in [20] to determine their mucin nature. Extracted material was subjected to Western blot. F1, F2 and F3 refer to the different purification fractions (for details see M&M). D) Neu5Az *trans*-sialylated trypomastigotes (900x10^6^) were lysed and sialylated proteins pulled-down with anti-FLAG antibodies. Western blots of this material were revealed with anti-TcMUC II antibodies. E-F) Confocal images displaying partial colocalization of anti-FLAG and anti-TcMUC II (E) or anti-αGal (F) labeling at the parasite surface.

The TS from living trypomastigotes accepts and readily sialylates the parasite surface with an azido-modified sialyl residue (Neu5Az) [6] from a donor that is α(2–3)-linked to a β-galactose such as (α2–3)azidosialyl-lactose (Neu5AzLac) or (α2–3)azidosialyl-β-galactose (Neu5AzGal) (Fig 1). The azido group is then covalently coupled to a phosphine-FLAG compound through a Cu^2+^-free click chemistry [22, 23], allowing to tag the transferred sialyl residue that, in turn, can be easily identified by anti-FLAG antibodies (Fig 1A). This unnatural sugar strategy allows analyze the labeling kinetics and fate of the sialyl residue on the parasite surface at the molecular level.

Following this approach, we supplied these sugar donors to live trypomastigotes to allow them to acquire the Neu5Az residue into their surface (Fig 1B) by means of the parasite TS. The Neu5Az residue was incorporated to diffuse bands between 60 and 190 kDa corresponding to the relative molecular mass for mucins of the trypomastigote stage [20]. To determine the mucin nature of the labeled material, we purified trypomastigote’s mucins following a standard butan-1-ol extraction protocol [8, 20]. Results shown in Fig 1C indicate that the sialylated material was isolated mainly in the F2 and F3 fractions, which are highly enriched in mucin-type glycoconjugates [8, 20]. The residual signal in F1, whose relative molecular masses coincide with that present in F2/F3, is consequence of a single extraction round. In agreement with that reported by Almeida et al [20], sialylated material and αGal containing mucins have identical purification patterns.

To further determine the sialylation of mucins, antibodies anti-FLAG were used to pull-down the Neu5Az-labeled material and retained and unbound fractions tested in Western blots using an antibody against the central and variable region of a defined *TcMUC II* product, a known trypomastigote mucin family [17]. As shown in Fig 1D, anti-TcMUC II reactivity was restricted to the bound, and thus Neu5Az-labeled fraction. In the same line, immunofluorescence experiments showed partial colocalization of TcMUC II and Neu5Az-labeling at the trypomastigote surface (Fig 1E). The relatively weak signal provided by the anti-TcMUC II antibody as compared to those yielded by anti-FLAG or anti-αGal (which recognizes the broadly distributed and immunodominant glycotope αGal) is consistent with the simultaneous expression of multiple TcMUC II products on the trypomastigote surface [17]. Despite this fact, results point to tGPI-mucins as main targets of the TS activity under our experimental conditions.

In search for another known surface marker of glycoproteins, we tested the αGal residue distribution. This residue is associated with mucin glycosylation although it is also present in other sialylated proteins such as Tc85 [27]. Given that sialylated and αGalactosylated proteins copurify in a mucin purification procedure, we were interested into analyze their display on the surface of the trypomastigote. Both Neu5Az and αGal displayed a dotted pattern but only partially colocalized. Given that Neu5Az and αGal are both linked to ßGal residues, this observation arises interesting questions regarding intracellular *vs*. extracellular mucin modifications. This finding also supports the presence of two types of mucins characterized by their glycosylation pattern and surface location.

**Fig 2:**
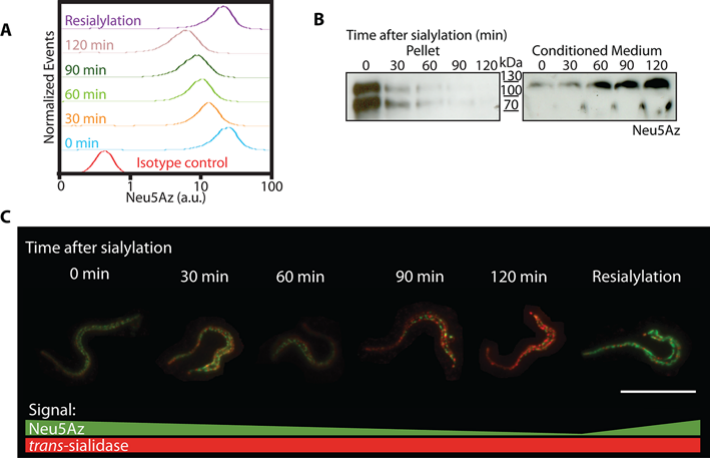
Shedding kinetics of sialylated mucins. Living parasites were incubated with Neu5Az donor. A) Acquired Neu5Az was followed by flow cytometry to determine its shedding kinetics. B) Western blot and C) immunofluorescence of the parasites from (A). Western blots indicate that Neu5Az was readily accepted into diffuse bands between 80-150kDa corresponding to the trypomastigote mucins [7]. The shed material was recovered in the conditioned media indicating that the residue was not hydrolyzed but conserved in the shed material. C) Fluorescence microscopy. Sialylated mucins fluorescence intensity fades while TS labeling remains. Raw images are shown to avoid biased observations. Bar: 10μm.

To analyze the shedding kinetics of Neu5Az-containing acceptors, live trypomastigotes were pulsed with Neu5AzLac and the shedding kinetics of sialylated mucins was followed by immunofluorescence, which was determined by flow cytometry in aliquots withdrawn every 30 minutes. Most of the acquired labeled sialic acid was lost after 120 minutes but, interestingly, parasites re-incubated with Neu5AzLac re-acquired sialic acid labeling to the saturating level (Fig 2A, Violet curve), indicating that sialyl acceptors had become available again. Relative molecular masses of the acceptor proteins were determined by Western blots of parasite lysates and conditioned media (Fig 2B). The sialyl residue was found associated to 80–150 kDa diffuse bands identified as trypomastigote mucins [20]. As labeled mucin signal faded on the parasite surface, it was increasingly recovered in the conditioned media indicating that the sialyl residue was not hydrolyzed but conserved in the shed material. Thus, the acquisition of new labeling indicates that *de novo* synthesized acceptor proteins have reached the parasite surface (and became available to the TS activity). Immunofluorescence microscopy imaging of the shedding process (Fig 2C) was in line with flow cytometry and Western blot findings. Sialic acid acceptors followed a dotted pattern distribution along the trypomastigote surface (see also below) that fades in keeping with the shedding kinetics. Parasites allowed to re-sialylate after shedding recovered their original labeling pattern. We found no special region or compartment of the cell surface primarily associated with the shedding process or morphological differences throughout the aliquots taken. TS staining was used as reference due to its continuous reposition to the trypomastigote surface [28]. Similar results indicating that no significant desialylation was involved were obtained in supporting assays where the only sialidase present, *i*.*e*. the TS activity, was neutralized by the addition of mAb 13G9 [29] after the labeling period.

Therefore, mucins have a constant turnover on the parasite membrane being replaced by *de novo* synthesized unsialylated proteins that acquire the sialyl residues as they become exposed on the surface to the sialyl donors and TS. Mucins half-life was estimated at 45 min with an almost complete turnover in about 2 hours.

### Sialylated mucins and TS are spatially segregated and show a spotted pattern along the trypomastigote membrane

**Fig 3:**
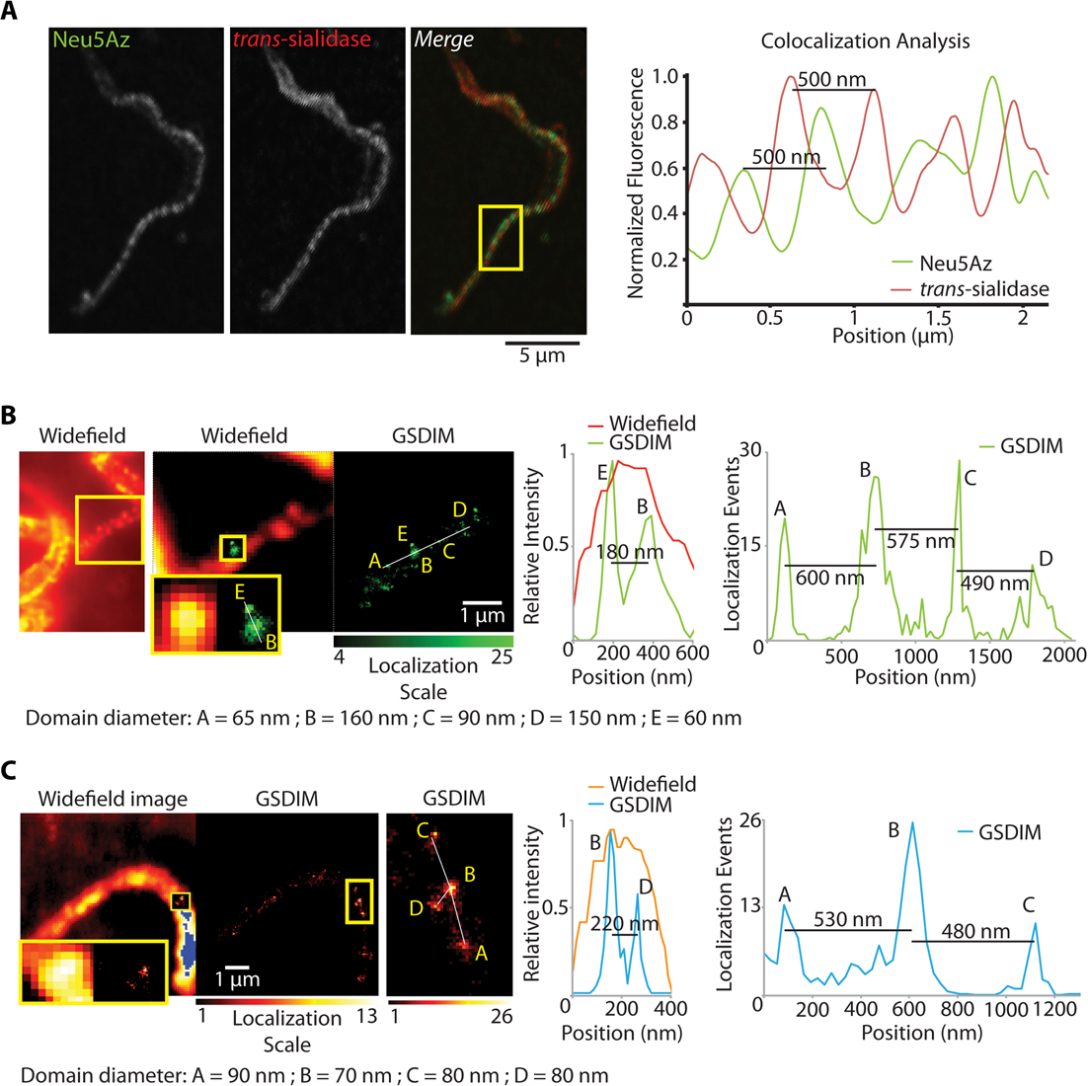
Sialylated mucins and TS are included in segregated membrane microdomains. A) Live trypomastigotes were sialylated and FLAG-tagged. Sialylated mucins and TS rendered a dotted pattern on the trypomastigotes surface that did not co-localize (confocal microscopy). Line profiles for sialic acid and TS signal in the flagellum (boxed area) showed that mucins and TS do not co-localize but were rather out-of-phase with each other. GSDIM superresolution fluorescence microscopy performed for sialylated mucins (B) and TS (C) independently showed that mucins were included in domains 90 nm wide and separated 120-500nm from each other. Results for TS were equivalent. Size and distribution of trypomastigote membrane domains explain why under a confocal microscope, restricted to classical light diffraction limits, the domains for TS and mucins were not fully resolved.

Live trypomastigotes were supplied with the Neu5AzLac donor and the acquired residue was revealed with Dibenzocyclooctynes-FLAG (DBCO-FLAG), which increment labeling kinetic rates by about 100-fold compared to phosphine reagents [30, 31]. Mucins were displayed in a dotted pattern following an unforeseen distribution localized both in the cell body and flagellum (Fig 1). As well as mucins, TS labeling followed a dotted pattern distributed along the flagellum and the cell body [6, 29]. It can be expected that the enzyme would be located close enough so as to allow the mucin sialylation to proceed. Notably, there was no colocalization between TS and Neu5Az signals (Fig 3A). Taking into account that TS and mucins are not only enzyme and substrate but also GPI-anchored proteins, their segregated distribution at the parasite surface is certainly puzzling. To ensure that the pattern was not an artifact due to the fixation process, a panel of several fixatives and temperatures was assessed and no differences were found.

Given the dimensions of the domains found for mucins and TS, we used a far-field superresolution fluorescence microscopy technique known as ground state depletion imaging (GSDIM) [32], a suitable tool to analyze phenomena that occur at the nanometric scale. This technique is based on the switching of most molecules from a conventional fluorophore to a metastable dark state, followed by the stochastic spontaneous return of a few molecules to the ground state where their position can be accurately estimated (Fig 3). The mucin domains were found to be a heterogeneous population with a mean diameter of 90nm and a separation of about 200-500nm between them. In addition, domains separated 60nm apart were found and could be resolved (Fig 3B, box). Domains for TS were also a heterogeneous sized population and similar mean diameters and distances between domains were recorded. Both for mucins and TS the distances between domains were equivalent to those determined by confocal microscopy (see Fig 3). However, GSDIM provided a 10-fold higher resolution that allowed determining accurate domain sizes (3-fold smaller than calculated by confocal microscopy) and spaces between them, enough for many others to be interspersed. These results provide further evidence that domains for mucins and TS do not colocalize and thus shed TS is responsible for mucin sialylation.

### Sialylated mucins are included in Lipid Rafts

**Fig 4:**
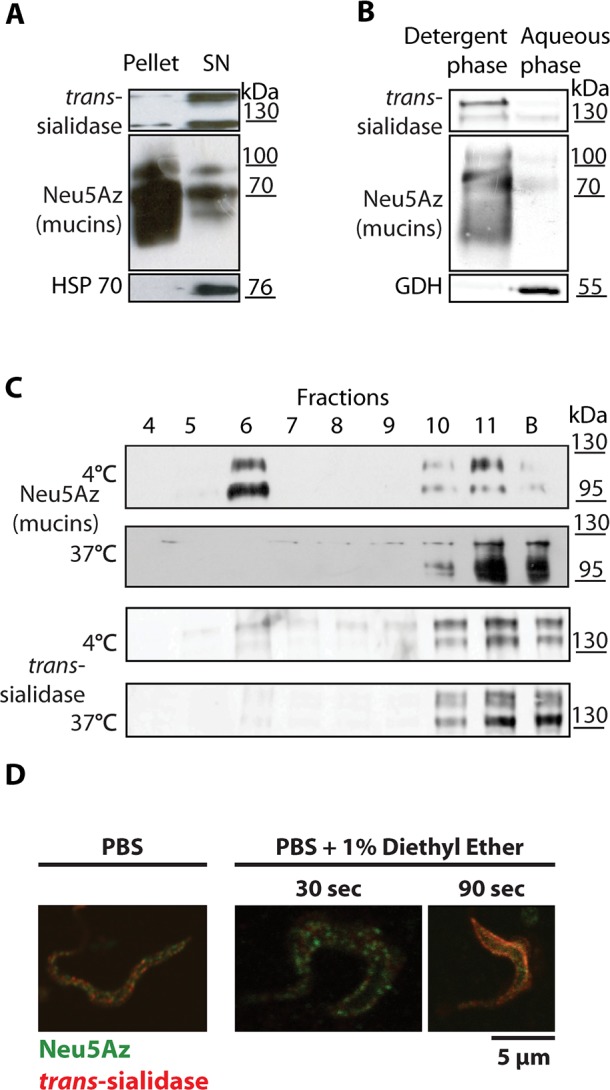
Sialylated mucins are included in lipid rafts whereas TS is not. (A) Cold Triton X-100 partition. Sialylated trypomastigotes were lysed at 4°C. Mucins were predominantly recovered in the pellet whereas TS and HSP70, a cytosolic protein, were recovered in the supernatant. (B) Triton X-114 extraction for GPI-anchored proteins. Parasites were lysed at 4°C and detergent and aqueous phases separated at 37°C and analyzed by Western blots. Mucins and TS partitioned in the detergent phase due to their GPI-anchoring. Glutamate Dehydrogenase, a cytosolic protein, was recovered in the aqueous phase. (C) Purification of DRMs by step-gradient ultracentrifugation. Trypomastigotes were lysed in Triton X-100 at 4°C or 37°C and centrifuged in an Optiprep gradient. Mucins floated to the 35%-5% interface (lane 6) only when lysis was done at 4°C indicating its DRM nature in contrast to TS. (D) Living parasites were sialylated from a Neu5Az donor, then treated for membrane fluidization with 1% diethyl ether in phosphate-buffered saline (PBS) and fixed with *p*-formaldehyde (PFA). Doted labeling for TS and mucins was disrupted only after 90sec treatment even showing colocalization.

The fact that sialyl-decorated mucins and TS were placed in discrete spots on the membrane of trypomastigotes lead us towards their possible inclusion in specialized membrane domains. Lipid rafts, or detergent resistant membranes (DRM) are a membrane organizing principle in which sterols, sphingolipids and glycolipids coalesce together to form membrane nanodomains with relative lower fluidity than the surrounding lipid bilayer [33]. Given their chemical composition, these domains tend to be enriched in GPI-anchored proteins and *trans-*membrane proteins that are sorted into these thicker membrane domains to maximize hydrophobic interactions [34]. Lipid rafts have already been described in several other protozoa [24] and in *T*. *cruzi* epimastigotes [25], but not yet in trypomastigotes. To address whether TS and mucins could localize to lipid rafts, cold Triton X-100 partition of Neu5Az-labeled trypomastigotes was performed. Fig 4 shows that, while mucins were recovered in the pellet, TS was present in the supernatant indicating mucin resistance to cold Triton X-100 extraction (Fig 4A). As a control, we also ran a Triton X-114 extraction. This detergent is miscible in aqueous solutions under 23°C and separates in a dense phase above that temperature making it suitable to extract GPI-anchored and lipophilic proteins. As expected, TS and mucins were recovered in the detergent phase likely due to their GPI-anchored nature (Fig 4B). To purify DRMs, pulsed trypomastigotes were lysed with Triton X-100 either at 4°C or 37°C and centrifuged on a discontinuous OptiPrep gradient. Neu5Az-labeled- mucins floated to the DRM fraction when lysis was performed at 4°C but were found at the bottom of the gradient together with other cell material when parasites were lysed at 37°C, temperature at which DRM are susceptible to detergent disruption (Fig 4C). Interestingly, TS did not float to the interface under any condition, indicating its exclusion from DRMs. These data support that the chemical environment in which mucins are located is different to that of TS, and provides independent evidence that mucins and TS are segregated at the trypomastigote surface.

To further study the properties of lipid rafts of trypomastigotes we assayed the effect of methyl-β-cyclodextrins [35], which severely altered the parasite structure (S1 Fig) and was therefore discarded as a tool to analyze lipid rafts. To visually test for the presence of lipid rafts, the trypomastigote membrane was fluidized with diethyl ether [35]. Neu5Az-sialylated parasites were exposed to diethyl ether for 30 or 90 seconds, immediately fixed and analyzed by immunofluorescence. As shown in Fig 4D, membrane domains were still conserved when parasites were exposed to diethyl ether for 30sec. However, after a 90sec exposition, sialic acid labeling was no longer localized uniquely at discrete membrane domains being rather uniformly distributed and even colocalizing with TS. This finding also indicates that the spotted pattern was neither due to capping by intermolecular cross-linking by antibodies nor to the fixation process, and supports that the fluidization of the parasites membrane disrupted the lipid rafts containing the sialylated mucins as well as the TS domains. These findings suggest the previously unnoticed existence of membrane rafts in *T*. *cruzi* trypomastigotes and highlight the possible relevance that membrane compartmentalization might have in the parasite biology.

### Mucin distribution in epimastigote and metacyclic trypomastigote surface

The distribution of mucins in the other parasite stages was analyzed following the same labeling strategy. Metacyclic trypomastigotes (the epimastigote-derived infectious form) were assayed under the same conditions used for cell culture-derived parasites and displayed similar distribution pattern (S2 Fig). Although epimastigotes (the replicative form found in the insect vector) do not express TS, their mucins are efficiently sialylated by adding the soluble enzyme in the presence of donors [36]. Although notoriously smaller in dot size [36], epimastigote sialic acid staining displayed similar distribution all along the cell body and flagellum (S2A Fig). Epimastigote mucins floated together with the lipid rafts when the Triton X-100 extraction was performed at 4°C but were found at the bottom when lysed at 37°C indicating that they were also included in lipid rafts. Western blots analysis of the Optiprep gradients displayed diffuse bands between 35 and 60kDa, the expected mass of this stage-specific mucins [10] (S2B Fig).

**Fig 5:**
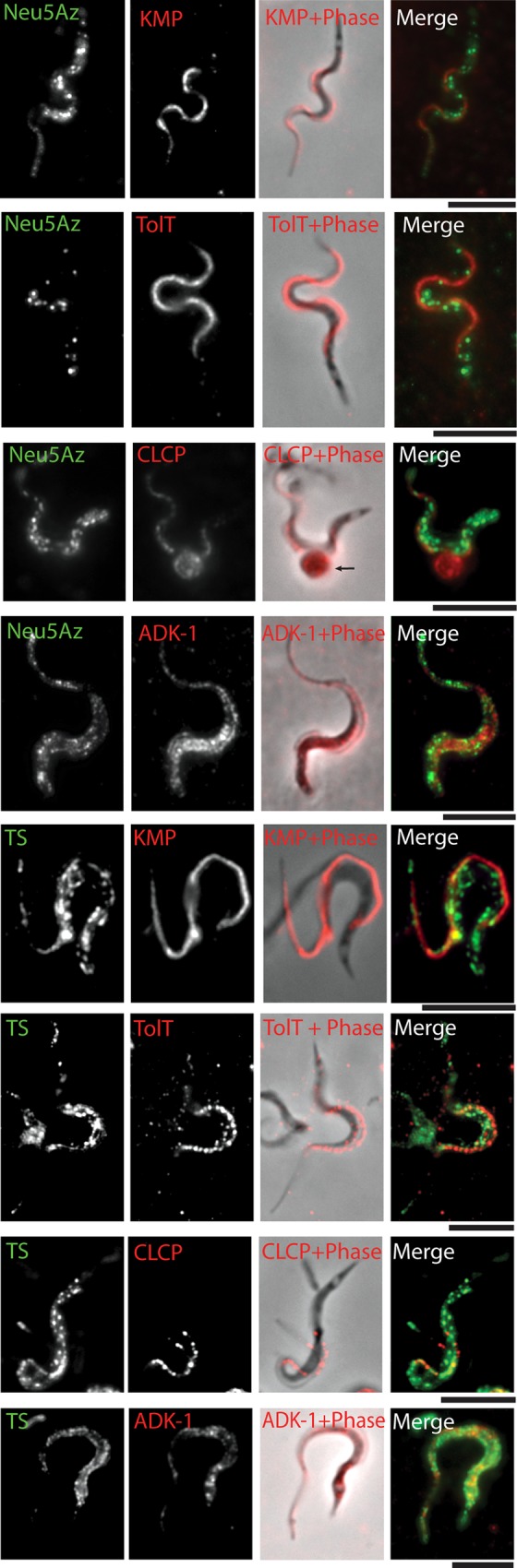
Distribution of proteins contained in DRMs. Proteins identified by mass spectrometry were analyzed by immunofluorescence. KMP, TolT and CLCP were located in domains of the flagellum. ADK-1 displayed a dotted pattern in the cell body and in the flagellum. No co-localization with mucins or with TS was found. Arrow points to an amastigote, this stage remains unsialylated. Bar: 5μm.

Amastigotes (the replicative intracellular stage in mammals) were collected from infected cell-cultures and, due to the absence of TS expression in this stage, were tested for sialic acid labeling by addition of recombinant TS and Neu5Az donors. However, no sialic acid labeling at all was detected even though stage-specific mucins have been described [37]. The absence of TS-available terminal ßGal residues in amastigote mucins (see Fig 5, panel Neu5Az/CCLP for an unsialylated amastigote) agrees with the absence of TS-suitable sialylated donors in the cytoplasm of mammalian cells. The unnecessary expression of TS in this intracellular stage seems to be an energy wasting process.

### DRM-domains of *T*. *cruzi* are heterogeneous in size population and are highly specific in their protein composition

To determine the protein composition of *T*. *cruzi* trypomastigote lipid rafts, mass spectrometry (LC-MS-MS) was performed on trypsin-treated samples obtained after two-tandem raft purification steps by discontinuous Optiprep gradient. Table 1 lists the most representative membrane proteins identified (S1 Table lists all identified proteins).

**Table 1:** Selected Hits from Mass Spectrometry Assays from Lipid Rafts Proteins.

Accession number	Protein description	References
407410164	small G-protein, putative Rab23	[38]
37727511	adenylate kinase 1 (ADK-1)	[39]
71402301/ 71401264/ 71401268	surface protein Tol-T	[40]
6166388	surface protein KMP11	[41, 42]
4234959	surface protein Tol-T3	
71407177	inositol 1,4,5-trisphosphate receptor	[43]
407845415/ 407391460	surface protein GP85	[44]
407831276/ 407413084	mucin-associated surface protein (MASP), putative	[45, 46]
71407848/ 71664661	calpain-like cysteine peptidase (CLCP)	[46, 47]

Two-tandem discontinuous Optiprep gradient ultracentrifugation was performed to highly purify DRMs. The resulting material was analyzed by LC-MS-MS. The most representative membrane proteins identified are listed together with their Gene DB accession number. For a complete list of identified proteins see S1 Table.

The proteins listed in the table had already been demonstrated to be membrane proteins and some were even found in the secretome of *T*. *cruzi* [46]. To validate the results obtained, we selected several of these identified proteins and analyzed their surface distribution by fluorescence microscopy. Recombinant Calpain-like cysteine peptidase (CLCP) [47], Kinetoplastid membrane protein-11 (KMP-11) [41, 42] and Tol-T [40] were expressed in bacteria to elicit antibodies in mice. Immunofluorescence assays (Fig 5) determined that these proteins were also distributed in discrete membrane domains, indicating a correlation with lipid rafts purification. Domains were smaller in size and mostly distributed in a linear pattern along the flagellum. Furthermore, none of these proteins colocalized with sialylated mucins or TS. We then tested Adenilate Kinase-1 (ADK-1) [39] that also exhibited a dotted pattern distributed both in the cell body and flagellum (Fig 5). Once again, no colocalization was observed between ADK-1 and mucins or TS. These results strongly support a segregation of different proteins along different membrane domains at the cell surface.

**Fig 6:**
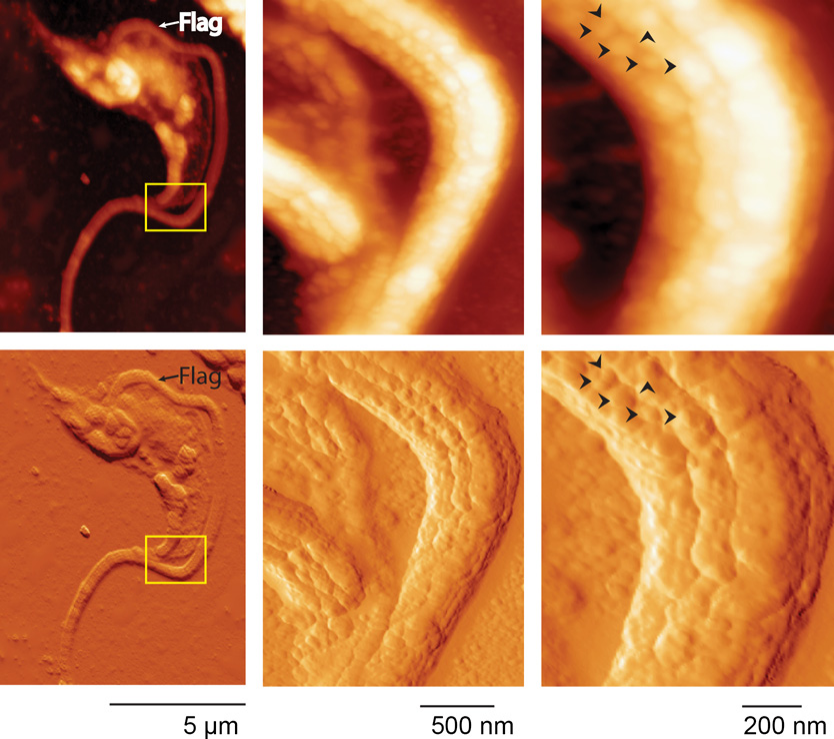
Atomic force microscopy (AFM). Trypomastigotes were AFM imaged by tapping mode. Upper panels show height traces and bottom panels show 3D transformation. Special focus was done over the flagellum where heterogeneous and irregular domains following parallel structures along the flagellum could be observed. Arrows indicate neighboring domains to highlight size distribution and domain separation. Flag: flagellum.

The puzzling non-colocalizing patterns found for the different proteins analyzed, together with other reports of trypomastigote proteins that also displayed dotted patterns [18, 25, 48] suggest a particular membrane topology with defined domains. To obtain highly solved topological information, we then assayed atomic force microscopy (AFM), a suitable tool that has been used in epimastigotes [49, 50]. AFM imaging of trypomastigotes showed defined protrusions on their surface, consistent with the presence of microdomains (Fig 6). These knobs could be visualized along the entire cell body and especially at the flagellum. These structures were heterogeneous in shape and size, from 10nm to 90nm wide, and narrowly packed together. Along the flagellum, they were displayed in parallel rows in an order that could suggest their association with the cytoskeleton. Findings disclosed by AFM were also consistent with those observed by GSDIM for both mucins and TS, thus providing topological evidence supporting the distribution of these different molecules in intercalated and tightly packed domains.

### TS and mucins are shed in microvesicles rather than via GPI-anchor hydrolysis

**Fig 7:**
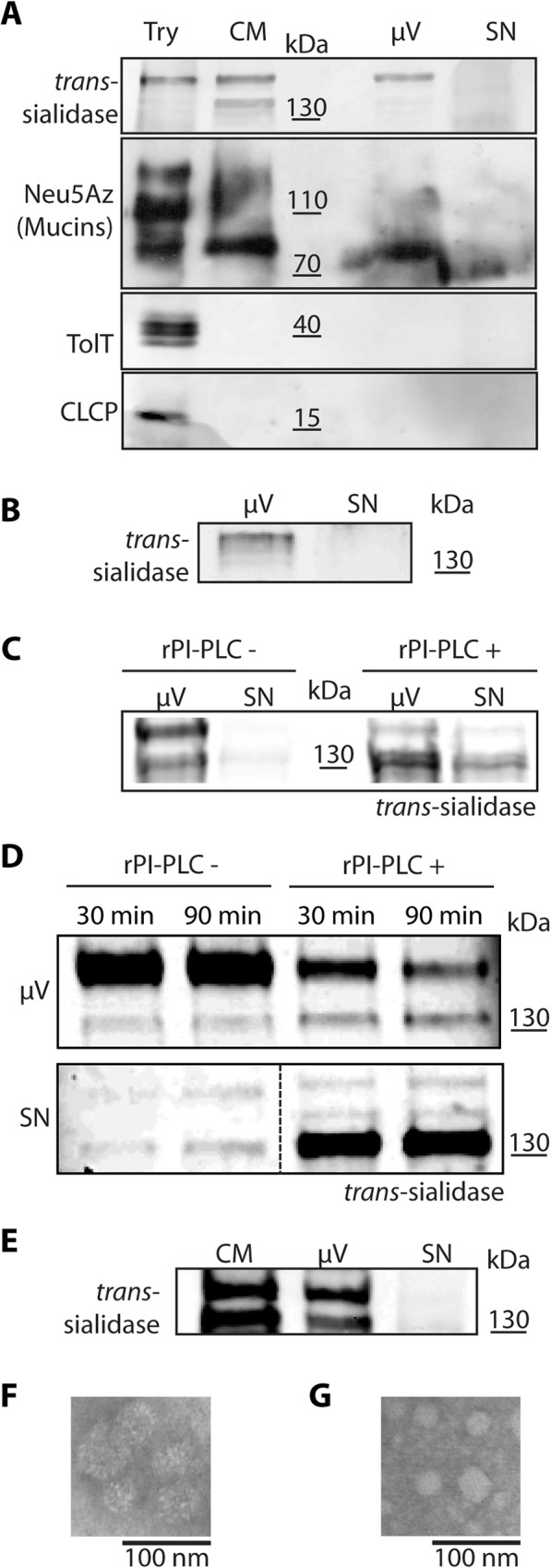
*trans*-Sialidase is shed in microvesicles. Western blots of microvesicles purified by two independent methods, Exoquick kit (A) and ultracentrifugation (B), show TS presence only in the microvesicles fraction. Mucins were found predominantly in microvesicles although also to a minor extent in the supernatant. TolT and CLCP proteins contained in DRMs, were not found in the conditioned media (A). TS was hydrolyzed by the addition of recombinant PI-PLC (rPI-PLC) from microvesicles (C) and from trypomastigotes (D) confirming the absence of endogenous PI-PLC. TS was also found in microvesicles from the supernatant of infected cell cultures evidencing no involvement of PI-PLC activity from Vero cells or amastigotes (E). F) and G) are TEM micrographs of microvesicles purified by Exoquick and ultracentrifugation respectively. Try; trypomastigotes, CM; conditioned medium, μV; microvesicles; SN; supernatant.

It has been reported that enzymatically inactive components of the TS superfamily are found in plasma membrane-derived microvesicles that are continuously shed by the parasite and that modulate infection via poorly understood mechanisms [15, 46]. To analyze whether TS and mucins are shed as soluble proteins or included in microvesicles, Neu5AzLac-pulsed trypomastigotes were rinsed and incubated in serum-free RPMI with DBCO-Biotin. After 3 hours microvesicles were purified with Exoquick from the conditioned medium and analyzed by Western blot. Fig 7A shows that TS was shed exclusively in microvesicles and mucins were distributed mainly in the microvesicle fraction. Similar results were obtained when microvesicles were collected from trypomastigote-conditioned media by ultracentrifugation (Fig 7B). In contrast, CLCP and TolT were not secreted by trypomastigotes (Fig 7A).

TS shedding in microvesicles rather than in a soluble form advocates against the hydrolysis of the GPI-anchor by PI-PLC as has been proposed [28, 37, 51]. In support, it is noteworthy that PI-PLC is not detected in trypomastigotes [52]. To analyze the relation between PI-PLC and TS shedding we tested whether the hydrolysis of the GPI anchor shifts the TS distribution from the microvesicle pellet to the supernatant. Digestion with recombinant PI-PLC was performed before purifying the microvesicles (Fig 7C) or PI-PLC was added to trypomastigotes in serum-free RPMI (Fig 7D). In both experimental set-ups, PI-PLC addition shifted TS localization from the vesicles to the soluble fraction, thus strongly supporting that there is no parasite-derived PI-PLC activity. Finally, to test whether differentiating amastigotes could be shedding enough PI-PLC to digest the TS-GPI anchor, we tested for the presence of soluble TS in the conditioned medium of an infected Vero cell culture (Fig 7E). Again, we only found TS associated to microvesicles rather than as a soluble protein. Thus, even if differentiating amastigotes shed PI-PLC [53], the enzymatic activity is not enough to quantitatively hydrolyze the TS GPI-anchor.

As a whole, our results support that the TS is shed from the trypomastigote surface included in microvesicles rather than in a soluble form after endogenous PI-PLC digestion. In addition to the physiological relevance of this finding, TS becomes a suitable marker for these secreted vesicles.

## Discussion

The glycobiology, and membrane dynamics of *T*. *cruzi* trypomastigotes are almost unknown due to the scarcity of assays on membrane biology of this infective parasite stage. Here we investigated two of the most relevant surface proteins that interact with the mammalian host during the infection. Mucins and TS are substrate and enzyme respectively in the sialylation interplay with a central role in the transference of the sialyl residue from the host. The unnatural sugars approach allowed us to address multiple experiments with unnoticeable chemical perturbation and high selectivity to the recently transferred sialic acid.

The kinetics of sialyl residue acquisition and the turnover of the sialylated mucins together with their surface distribution were addressed. A striking finding was that TS and its mucin substrate do not colocalize at the parasite surface. Trypomastigotes glycoconjugates distribution had been previously addressed by ruthenium red staining [8], a dye used for electron microscopy detection of saccharides. Authors found a dense coat of labeling that was interpreted as a uniform distribution of mucins. However here we found that mucins, either sialylated or α-Galactosylated, were actually distributed in patches instead (Figs 1 and 2).

The linear repetitive SAPA extension of the TS acts as a spacer that separates its catalytic domain from the membrane [28, 51]. Depending on the *T*. *cruzi* strain, TS isoforms may be expressed with even more than twenty repetitive units (of a motif of twelve aminoacid residues each). A SAPA extension of twelve repetitive units will avert the TS catalytic domain to about 50nm away from the cell surface. The combination of confocal and superresolution microscopies used here determined that domains for mucins and TS should be separated apart by about 150nm. Due to this spatial distribution, membrane anchored TS becomes unable to sialylate mucins. Independent biochemical evidence is presented to support the segregation of these two kinds of molecules on the trypomastigote surface. These proteins have shown via detergent susceptibility to be anchored to microdomains with different lipidic environments. Thus, we speculated on alternatives of how mucins might become sialylated by TS: (i) newly synthesized mucins could go through a TS-rich area when passing through the flagellar pocket to the surface thus becoming immediately sialylated or (ii) mucins become sialylated by the TS shed to the extracellular environment. To study the first hypothesis, we followed the sialylation of newly synthesized mucins. These were neither found colocalizing with TS nor associated to a specific region of the parasite (see S3A Fig). These results support that newly exposed mucins haven’t been in close contact with TS all along their path to the surface. To obtain direct evidence of the ability of the shed TS to label the surface of distant parasites, we incubated PFA-fixed trypomastigotes with live parasites. Fast acquisition (1 min) of sialyl residues was observed, consistent with the involvement of the shed TS in this process (S3B Fig). The hypothesis associating the shed TS with mucins sialylation is also supported by the fact that TS is rapidly detected in trypomastigote-conditioned supernatants [6] as well as in the cytoplasm of infected cells loaded with trypomastigotes [54]. Thus, *in vivo* when infected cells burst, high amounts of TS and trypomastigotes become in contact with sialic acid donors from the extracellular milieu, leading to a rapid sugar acquisition by the parasites. The combination of nano-scale microscopic techniques such as AFM displaying membrane topology and GSDIM displaying specific protein distribution have shown how the membrane of *T*. *cruzi* is covered in microdomains which are 10nm to 90nm wide and packed close together. We have also shown by confocal microscopy how these membrane domains are forming a protein-specific patchwork, particularly concentrated in the flagellum. Therefore, not only are microdomains for mucins and TS separated apart but also between them there exists a dense patchwork of protein-packed microdomains, which presents an additional steric hindrance for the bound TS to access mucins. Analyzing the big picture, the hypothesis that membrane bound TS is unavailable to sialylate mucins finds further support.

The finding that two different mucin types based in their glycosylation pattern do exist in *T*. *cruzi* was also astonishing. From the initial studies it was observed that mucins are the main acceptors of sialyl residues [10]. In search of the proteins containing the biologically relevant αGal epitope, the lab from Dr. Almeida biochemically characterized them as mucins [20]. Organic solvents extraction rendered heavily *O*-glycosylated proteins containing αGal and sialyl residues [20]. We followed this purification protocol and parallel pull-down assays to demonstrate that the Neu5Az-sialylated proteins were actually tGPI-mucins (Fig 1). Particularly, sialylation and αGalactosylation are both possible modifications on terminal ßGal-bearing glycans and copurify by mucin extraction procedures. Interestingly, αGalactosylation occurs intracellularly whereas sialylation is an extracellular modification. Hence, a major question regarding mucin identity is whether the same mucin molecule can be both terminally decorated with αGal and sialyl residues. We present evidence of only partial co-occurrence of these residues in membrane domains. Our results indicate that sialyl and αGal residues could be in some cases linked to the same glycoconjugate and in other cases contained in different mucin populations. Whether they are representatives of different gene families or just different glycosylation of similar proteins are motive of future research. These unexpected findings add another degree of complexity to the parasite surface composition/structure. It is noteworthy that sialylation of parasites prevents their damage by anti-αGal antibodies present in chagasic patients [8]. However, the access of antibodies directed to αGal in sialylated parasites indicates that sialylation does not mask those epitopes.

The TS from *T*. *cruzi* is found in blood of acutely infected mammals. However, the exact way through which it is shed remained elusive. Due to its GPI-anchor, TS shedding through endogenous PI-PLC hydrolysis was proposed [28, 37, 51]. However, PI-PLC expression is developmentally regulated along the life cycle of *T*. *cruzi* and it is only expressed during the differentiation of trypomastigotes to amastigotes and just before the re-differentiation to trypomastigotes [53, 55] while in trypomastigotes it could not be detected [52]. In amastigotes, PI-PLC is exported to the plasma membrane by a process driven by two lipid anchors [56, 57]. The addition of an exogenous PI-PLC probed that the TS-anchor is already available to these enzymes (Fig 7). However, we found that TS was only shed in microvesicles instead. No TS remained in the supernatants after microvesicles depletion confirming that PI-PLC is not expressed in trypomastigotes as previously reported [52]. Pioneering works on TS reported that, by molecular exclusion chromatography, TS was found in fractions sized over 700kD. This finding was interpreted as protein oligomerization through the SAPA repeats based on the idea that the GPI anchor was hydrolyzed by an endogenous PI-PLC [28, 51]. It has also been reported that TS is shed in vesicles ranging 20-80nm consistent with the vesicle size described here [58]. In view of the results shown here, the observed molecular mass of the secreted TS could therefore be explained due to its association to vesicles. Thus the classical paradigm of TS shedding through hydrolysis of its GPI-anchor should be revisited.

**Fig 8:**
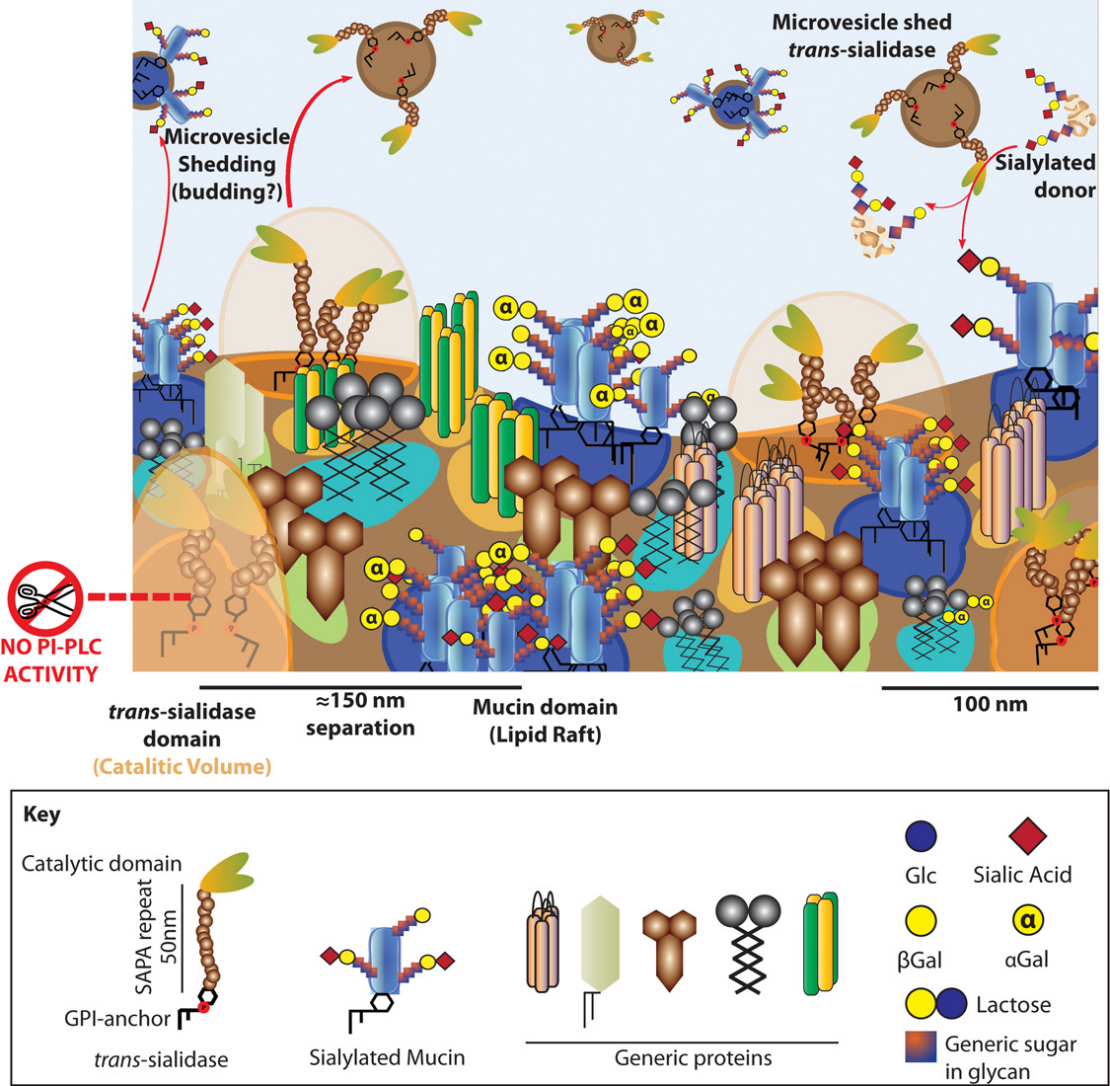
The membrane of trypomastigotes is complex to the nanometer scale. Membrane model for *T*. *cruzi* trypomastigotes. The surface is packed in microdomains of different size, shape, lipid composition and embedded proteins. Some of these domains are detergent resistant, however this does not imply a functional profile. Mucins are included in DRMs whereas TS is not, thus being segregated in the membrane of trypomastigotes. This challenges the membrane bound TS as the sialylating factor for mucins, a role proposed for the shed TS instead. DRMs embed different proteins, many of them localized to the flagellum. Flagellum domains tend to be smaller and closer together than those in the cell body and suggest an association to the flagellar cytoskeleton. Mucins and TS are shed to the extracellular environment included in microvesicles probably resulting from membrane budding and fission events. Furthermore, TS is shed associated to vesicles instead of as a soluble protein. No hydrolysis of the GPI-anchors occurs in the trypomastigote stage.

Trypomastigote surface seems to be rugous due to the presence of proteins [24, 59]. However, it constituted an overwhelming finding to realize that different classes of proteins are almost individually located in separate islets at the parasite surface. Different proteins tested were found located to different domains following a patchwork display. Some of them were clearly associated with the flagellum such as CCLP, TolT or KMP while others were more evenly distributed such as mucins, TS or ADK-1. It is interesting to compare results from *T*. *cruzi* with those from *T*. *brucei*. The flagellum of *T*. *brucei* is highly enriched with lipid rafts [35] that are smaller in size and not resolvable by light microscopy unless the membrane fluidity is tightened with dimethyl sulfoxide addition, a trick that was not necessary with *T*. *cruzi*. In this sense we have to recall that *T*. *cruzi* membranes contain mainly ergosterol instead of cholesterol, which is known to favor the production of highly stable DRMs in yeast [60]. It is interesting to observe a similar phenomenon when comparing lipid rafts in the flagellum and cell body of *T*. *cruzi* trypomastigotes. Whereas domains in the cell body (i.e. mucins, TS, ADK-1) are discrete, domains for flagellar proteins (i.e. CLCP, TolT, KMP) are smaller and barely resolvable, showing the same trend as for flagellar lipid rafts in *T*. *brucei*. From the results reported here it seems that, in *T*. *cruzi*, lipid rafts constitute an organizing principle by which proteins are specifically segregated on special areas of the surface as demonstrated in mammalian cells. Moreover, lipid rafts are not the only lipidic structure present in the trypomastigote membrane since other relevant proteins such as TS are included in detergent sensitive domains. We support the idea that each domain, regarding the specific proteins and lipids it bears, will present differential physicochemical properties [61] and will contribute to the orchestrated and variable membrane physiology of trypomastigotes along the biology of this *T*. *cruzi* stage. However, it still remains unknown how static or dynamic these domains are and their association with the cytoskeleton [62]. We hypothesize that some of these domains may serve as scaffolds for the formation of curved membranes that result in the budding of vesicles [63–66] while others or the same domains may also be important for the adhesion and invasion processes. It is important to establish the idea of an ordered and compartmentalized membrane in trypomastigotes and to establish which physiological events are associated to this ordered nature (see Fig 8 for a model). Through the integrated study of their glycobiology, *T*. *cruzi* trypomastigotes begin to unveil their membrane physiology.

## Materials and Methods

### Ethics statement

The protocol of animal immunization followed in this study was approved by the Committee on the Ethics of Animal Experiments of the Universidad Nacional de San Martín, according with the recommendations of the Guide for the Care and Use of Laboratory Animals of the National Institutes of Health

### Parasites

Tissue culture-derived trypomastigotes of the CL-Brener strain of *T*. *cruzi* were obtained from supernatant of infected Vero cells (ATCC, VA) cultured in Minimum Essential Medium (MEM) (Gibco, NY) plus 4% fetal bovine serum (FBS, Gibco) at 37°C and 5% CO_2_. Epimastigotes of the same parasite strain were axenically grown in LIT (Liver infusion-Tryptose) medium (Gibco).

### Staudinger-Bertozzi click chemistry for sialic acid labeling of acceptor molecules

To specifically label recently transferred sialic acid we used the Staudinger-Bertozzi (copper free) click-chemistry approach [22, 23]. Neu5Az-LacβOMe (Neu5AzLac) and Neu5Az-GalβOMe (Neu5AzGal) were synthesized [67]. The Neu5Az sialic acid was reacted with several compounds: Phos-FLAG, Phos-(PEG)_3_-Biotin (Thermo Scientific, Waltham, MA) DBCO-(PEG)_4_-Biotin and DBCO-FLAG (Jena Bioscience, Germany) [30, 31]. All compounds were used at saturating conditions. Equivalent labeling between compounds was verified by Western blot and immunofluorescence. Phos-FLAG or DBCO-FLAG were preferred for imaging because they showed significant lower background. To label the parasites with Neu5Az, they were incubated for 30min in PBS containing 1mM Neu5AzLac or Neu5AzGal as donor and 10mM 2-deoxi-Glucose (Sigma, MO). The enzymatic labeling was done by the endogenous parasite-derived TS in the case of trypomastigotes or by the addition of recombinant TS (10ng/10^6^ cells) for epimastigotes. After thoroughly washing, parasites were incubated overnight with Phosphine compounds or for 30min with DBCO compounds, which have faster reaction kinetics [30, 31]. As controls, trypomastigotes were incubated with 3’sialyllactose (Carbosynth, UK) and labeled through the same procedure.

### Recombinant protein expression and purification

Recombinant TS was expressed in bacteria and purified as described elsewhere [3]. CLCP [47], KMP-11 [41, 42] and Tol-T [40] encoding genes were cloned by PCR performed on genomic *T*. *cruzi* DNA and cloned into pTrcHis (Invitrogen) to be expressed in *Escherichia coli* BL21. Proteins were purified by immobilized metal affinity chromatography through Ni^2+^-charged Hi-Trap chelating columns (GE-Healthcare) to immunize BALB/cJ mice. The central and variable region (nucleotides 109 to 390) of one *TcMUCII* gene (GenBank U32448.1) was cloned by PCR into pGEX-1λT vector (GE Healthcare). GST-TcMUCII protein was expressed and purified by affinity chromatography and used for immunization. The protocol of animal immunization followed in this study was approved by the Committee on the Ethics of Animal Experiments of the Universidad Nacional de San Martín (UNSAM), according with the recommendations of the Guide for the Care and Use of Laboratory Animals of the National Institutes of Health.

### Mucin purification

Heavily *O*-glycosylated mucins were extracted from Neu5Az-*trans*-sialylated trypomastigotes with organic solvents as described in [20] and then the sialylation checked in extracted material by Western blots. Briefly, 900x10^6^ parasites were extracted with Chloroform/Methanol/water (5:10:4), centrifuged at 12,000Xg and pellet and extract dried under N_2_. The extract was treated with butanol/water (2:1) and centrifuged at 12,000Xg to separate butanolic (F1) and aqueous phase (F2). The pellet was extracted with 9% butanol and the 12,000Xg supernatant (F3) separated from the cell debris.

### Pull-down of sialylated proteins

Trypomastigotes (900×10^6^) were pulsed with Neu5Az donors as above, rinsed and coupled for 30min with DBCO-FLAG. Then parasites were resuspended in 300μl of IP buffer (150mM NaCl; 0,1%, Np40; 1% Triton X100; 50mM Tris-HCl pH7.6; Sigma protease inhibitor cocktail plus 50μM Tosyl-*L*-lysine Chloromethyl ketone hydrochloride (TLCK)) and lysed for 20min at RT followed by 40min on ice with occasional homogenization. The lysate was clarified by two centrifugation steps (15min; 10,000rpm; 4°C) and pellets discarded. An anti-FLAG (M2 clone) antibody coupled to a resin (Sigma) (25μl) was rinsed 4X with 500μl of IP buffer. One third of the clarified lysate was kept as the Input fraction and the other were pulled down with anti-FLAG antibodies, the supernatant was kept as the Unbound fraction. Resin was washed, cracked and processed for Western blots revealed with mouse antibodies anti-TcMUCII.

### Immunofluorescence microscopy

For immunofluorescence microscopy, 12mm coverslips (Marienfeld, Germany) were coated with 0.2mg/ml poly-L-lysine (Sigma), rinsed and air-dried. Trypomastigotes were fixed for 1h at 4°C with 4% EM grade PFA, (Electron Microscopy Sciences, PA) and settled for 1h on coated coverslips. Samples were blocked with 0.45μm-filtered 2% biotechnology grade-bovine serum albumin (BSA, Amresco, OH) in PBS. Either primary mAb or immune sera were diluted in blocking solutions. AlexaFluor488 and AlexaFluor568 coupled secondary antibodies (Invitrogen, IL) were used at a final concentration of 2μg/ml. Coverslips were mounted using FluorSave (Calbiochem, Germany). Epi-fluorescence imaging was done under a Nikon Eclipse E600 microscope equipped with a 100× NA:1.4 objective and with a Diagnostic Instruments SPOT RT Slider CCD camera. Exposure times were shorter than those in which no signal was recorded for negative control. Confocal imaging was done under an Olympus FV1000 microscope equipped with a 100× NA:1.4 or 60× NA:1.44 objective. Pinhole was set to obtain minimum-thick Z slices (0.8μm). Resolution was set to generously cover Rayleigh criterion. Maximum photo-multiplier HV parameter was determined with negative controls. The displayed images correspond to a single slice of the confocal stack. All images were taken at room temperature. Advanced microscopy techniques are described separately. Images and colocalization profiles were analyzed using ImageJ (NIH, Bethesda).

### GSDIM superresolution microscopy

#### Image acquisition

Single molecule images were acquired on a home-built wide field microscope with EMCCD (IXON-DU-897; Andor Technology) detection. The samples were continuously excited with 532nm laser diode (SDL-532-200T; Shanghai Dream Lasers Technology) and power density was controlled with a neutral density filter between 1kW/cm^2^ to 30kW/cm^2^. In order to achieve an optimal event number per frame, a 405nm laser (SDL-405-LM-20-T; Shanghai Dream Lasers Technology) was used to depopulate the dark states at a power density of 0.6kW/cm^2^. This laser was switched on and off using a homemade pulse generation unit connected to the fire signal of the EMCCD and the blanking signal of the lasers. In this manner, the sample was irradiated for short periods, preferable between frames. Both laser beams were collinearly overlapped (DCLP 425 (DCXR) AHF, and focused (Achromat f200 lens) onto the back focal plane of a Leica PLAN 100× objective, 1.25 NA, oil immersion, to achieve wide-field illumination. Single molecule fluorescence emission was collected by the same objective, and separated from the excitation beams by means of a dichroic mirror (Z488-532rpc; Chroma Technology). Emission filters were chosen for Alexa532 (562–40, Semrock Inc.) and Alexa568 (605–40, Chroma Technology) and placed in front of the detector. Excitation residual light was further cleaned with a notch filter (NF01-532U-25; Semrock Inc.). The pixel size of 83×83nm^2^ on the focal plane was calibrated with a resolution test pattern (USAF 1951 RES TARGET 2IN, Edmund Optics). Typically, sequences of images were recorded onto a 70×70 pixel region of the EMCCD camera (ca. 6×6μm). Image acquisition was performed with the software of the provider of the camera (Andor Solis; Andor Technology) at an exposure time of 50ms. XY in plane stability of the system was followed in time with fluorescent bead samples (TetraSpeck Microspheres, 100nm, blue/green/orange/dark red fluorescent, Life technologies). Typically, random fluctuations were smaller than 5nm. All measurements were performed at room temperature. Sequences of images were acquired consecutively till the number of events per frame was too low for SR imaging, thus a typical imaging experiment consisted of ca. 30,000 frames.

#### Imaging buffers

Imaging buffer contained TN buffer (50mM Tris, pH 8.0, 10mM NaCl), an oxygen scavenging system (0.5mg/ml glucose oxidase (Sigma), 40μg/ml catalase (Roche Applied Science; Sigma), and 10% (w/v) glucose (Sigma)), and 10mM 2-mercaptoethanol (Fluka, Germany). Imaging buffer was optimized for the system as described elsewhere [68].

#### Image processing software

Localization of single-molecule events and the superresolution image reconstruction were performed automatically by a series of homemade routines (written in Matlab Software) applied to the imaged stacks. Briefly, each frame was processed with a smoothing Gaussian filter and events were identified as a cluster of pixels (typically > 5) with intensity higher than a threshold, set as at least four times the standard deviation of the background signal. Then, the center of mass was determined for each cluster. The exact position of each cluster was determined with a procedure based on a Gaussian mask-fitting algorithm described in detail elsewhere [69, 70]. As an output, a list of localized events was constructed. The list contained the position, intensity in number of collected photons (based on a calibration over the camera detection) [71] and the frame where the event occurred. Events that appeared in consecutive frames at the same position (within an error of ca. 100nm, i.e. about one pixel) were considered as the same molecule and added to a single burst (photons were added and position recalculated). This analysis allowed estimating the mean on time of the fluorophores, data that was used to set an appropriate imaging frame rate. The final image was generated by plotting a 2D histogram of the list of positions rendered by the localization routine. The imaged area was reconstructed with a pixel size of about one half of the theoretical average final resolution (calculated from the distribution of the number of photons detected per localized event), and the intensity of each pixel was assigned as the number of single molecules localized within its area.

### Atomic force microscopy

Trypomastigotes were rinsed twice in PBS and fixed in 4% *p-*formaldehyde. Then, 10^6^ trypomastigotes were settled on poly-*L*-lysine treated coverslips, dehydrated by increasing ethanol concentration series and critical point dried. Images were taken using a Veeco Nanoscope III atomic force microscope in tapping mode under a N_2_ atmosphere. MPP-11100 cantilevers (Nanodevices, CA) with spring constant of 40N/m; f0 = 300Khz and r <10nm were used. Images were processed with WSxM software from WSxM solutions (www.wsxmsolutions.com).

### Mucin shedding kinetics

Trypomastigotes (150×10^6^) were pulsed for 30min with Neu5AzLac as described above, rinsed and resuspended in RPMI 1640 medium (Gibco). Trypomastigotes were aliquoted and incubated at 37°C in 5% CO_2_. Every 30min an aliquot was withdrawn, centrifuged and the pellet separated from the conditioned medium. After 120min, one aliquot was re-pulsed with Neu5AzLac for 30min. For immunofluorescence assays, parasites were fixed as above, rinsed and incubated overnight with Phos-FLAG. Fluorescence intensity was quantified by flow cytometry. For Western blot analysis, parasites were lysed in Lysis buffer (150mM NaCl, 1% Triton X-100, 50mM Tris-HCl pH 7.6, 1X protease inhibitor cocktail (Sigma), 50μM TLCK and 13G9 TS-neutralizing mAb) [29], then incubated overnight with Phos-FLAG at 4°C. Conditioned media were supplemented with 1X protease inhibitor cocktail, 50μM TLCK plus 13G9 mAb and then incubated with Phos-FLAG overnight at 4°C. Western blots from pellets and conditioned media from 10% SDS-PAGE were developed with a rat anti-FLAG mAb (Biolegend, CA) using a chemioluminescent substrate (Thermo Scientific, IL).

### Differential Neu5Az uptake

Trypomastigotes (150×10^6^) were pulsed for 30min with 3’sialyllactose, rinsed, aliquoted and reincubated with Neu5AzLac for 30sec to 15min. Then, parasites were fixed immediately in 4% PFA. All aliquots were rinsed twice and incubated overnight with Phos-FLAG. Immunofluorescence assays were conducted against FLAG and TS.

### Sialylation of trypomastigotes by shed TS

Trypomastigotes where labeled with mouse mAb 13G9 at saturating conditions, washed and fixed with PFA. Fixed trypomastigotes were exhaustively rinsed and resuspended to 160×10^6^ parasites/ml and Neu5AzGal was added to 1mM. Several 25μl-aliquots containing 4×10^6^ parasites were prepared. Separately, live trypomastigotes were rinsed and resuspended to 160×10^6^ parasites/ml and 25μl were added to each aliquot of fixed parasites. At given times, co-incubation was stopped by addition of an equal volume of 4% PFA. As a negative control, an aliquot was added with 4% PFA before addition of the live trypomastigotes. As positive control 8×10^6^ fixed parasites were incubated with 250ng of recombinant TS for 30 min. To label Neu5Az residues, parasites were rinsed and incubated overnight with PhosFLAG. Rat anti-FLAG antibodies and anti-Rat Alexa488 secondary antibodies were used for Neu5Az staining and anti-mouse Alexa568 secondary antibodies were used to identify mAb 13G9-labeled parasites.

### Triton X-100 assay for DRM

Trypomastigotes (100×10^6^) were pulsed for 30min with Neu5AzGal as described above, rinsed and lysed in 100μl of lysis buffer at 4°C for 30 min. Then, the lysate was centrifuged at 10,000xg in an SS34 rotor at 4°C. Pellets and supernatants were stored at -20°C overnight. Pellet was resuspended in 100μl lysis buffer (added with protease inhibitor cocktail (Sigma) plus 50μM TLCK and 13G9 mAb) and Phos-FLAG. The supernatant was supplemented with protease inhibitor cocktail (Sigma), 50μM TLCK, 13G9 mAb and reacted with Phos-FLAG. Samples were incubated overnight at 4°C cracked and ran in a 10% SDS-PAGE to perform Western blots.

### Triton X-114 assay for GPI-anchored proteins

Trypomastigotes (100×10^6^) were pulsed for 30min with Neu5AzGal as described above, rinsed and reacted with DBCO-Biotin for 30min. Then parasites were lysed for 1h at 4°C in 2ml Triton X-114 extraction buffer (150mM NaCl, 2% Triton X-114, 50mM Tris pH 7.6, protease inhibitor cocktail (Sigma) and 50μM TLCK). Lysate was stored at -20°C overnight. Then, the lysate was incubated for 10min at 37°C and centrifuged at 3,000g for 10min at room temperature. Detergent and aqueous phases were collected and precipitated overnight with 3 volumes of cold acetone at -20°C. Precipitates were cracked to perform Western blots.

### Step gradient purification of DRMs

Trypomastigotes (800×10^6^) were pulsed for 30min with Neu5AzGal as above, rinsed and separated in half. Each aliquot was lysed in 1ml of lysis buffer supplemented with DBCO-Biotin either on ice or at 37°C for 45 min. Then, lysates were deposited on the bottom of ultracentrifuge tubes and adjusted to 40% OptiPrep by adding 2ml of a 60% OptiPrep solution (Sigma). On top of the 40% OptiPrep layer, 5ml of a 35% OptiPrep solution was settled. Finally, the tube was filled with a layer of 5% OptiPrep. All OptiPrep solutions were fixed to 150mM NaCl, 50mM Tris pH 7.6 and 1% Triton X-100. Samples were then centrifuged in a Beckman Optima XL-100K ultracentrifuge using an SW41Ti rotor. Samples were continuously centrifuged at 35,000rpm 210,053×g (max) for 5h and then at 25,000rpm 107,170×g (max) for 12h at 4°C. Finally, 1ml aliquots were withdrawn top to bottom except for the two interfaces, which were collected in 300μl to avoid dilution and contamination. Fifty μl of each aliquot were cracked for Western blot.

### Mass spectrometry of DRMs

DRMs were prepared for MS by two consecutive step-gradient purifications as described above. Trypomastigotes (800×10^6^) were lysed and centrifuged as above. The 35%/5% OptiPrep interface was collected in 1ml into an ultracentrifuge tube, mixed with 60% OptiPrep to a final 40% OptiPrep concentration and purification continued as before. After the second centrifugation step, the 35%-5% OptiPrep interface was collected in 300μl to avoid contamination and precipitated with 3 volumes of cold acetone. Samples were analyzed at ITSI-Biosciences (Johnstown, PA) by in solution digestion and tandem mass spectrometry analysis using nano-LC/MS/MS. After digestion, peptides were dried and reconstituted in 2% Acetonitrile with 0.1% Formic acid. After salt removing, samples were loaded onto a PicoFrit C18 nanospray column (New Objective) using a Thermo Scientific Surveyor Autosampler operated in the no waste injection mode. Peptides were eluted with a 2–32% linear Acetonitrile gradient over 230 minutes followed by high and low organic washes for another 5 min into an LTQ XL mass spectrometer (Thermo Scientific) via a nanospray source with the spray voltage set to 1.8kV and the ion transfer capillary set at 180°C. A full MS scan from m/z 350–1500 was followed by MS/MS scans on the seven most abundant ions. Raw data files were searched using Proteome Discoverer 1.3 (Thermo Scientific) and the SEQUEST algorithm against the most recent species-specific database for *T*. *cruzi* from NCBI. Trypsin was the selected enzyme allowing for up to two missed cleavages per peptide, Carbamidomethyl Cysteine was used as a static modification and Oxidation of Methionine as a variable modification. Proteins were identified when two or more unique peptides had X-correlation scores greater than 1.5, 2.0, and 2.5 for respective charge states of +1, +2, and +3. A total of 152 proteins were identified.

### Membrane fluidity assay

Trypomastigotes were pulsed for 30min with Neu5AzLac as above, rinsed and settled over poly-*L*-lysine (Sigma)-treated coverslips for 10min. Then, the coverslips were inverted on freshly prepared solutions of PBS or PBS plus 1% diethyl ether. Coverslips were incubated for 30, 60 or 90sec and immediately inverted over 4% PFA. Coverslips were then processed for immunofluorescence as above.

### Microvesicle harvest of trypomastigote conditioned media

Trypomastigotes (200×10^6^) were pulsed for 30min with Neu5AzLac, rinsed and incubated in 200μl of FBS-free RPMI-1640 plus DBCO-Biotin for 90min at 37°C and 5% CO_2_. Parasites were separated by four consecutive centrifugation rounds, two at 2700×g for 10min followed by two centrifugations at 10,600×g for 10min. The cell-free conditioned medium was supplemented with 10% FBS to a final volume of 100μl. Exosome purification kit (System Biosciences, CA) was added to the conditioned medium and microvesicles were purified according to the manufacturers protocol. Briefly, the Exoquick supplemented conditioned medium was allowed to settle at 1g overnight and then centrifuged 30min at 1,500xg. The pellet containing microvesicles was cracked in a final volume of 100μl while proteins in the supernatant were precipitated with 3 volumes of cold acetone, stored at -20°C overnight and centrifuged at 13,000g for 30min. Precipitated proteins were cracked in a final volume of 100μl and developed by Western blot.

### Microvesicle harvest by ultracentrifugation

Trypomastigotes (240×10^6^) were rinsed and incubated in 10ml of FBS-free RPMI-1640 for 90min at 37°C and 5% CO_2_. Trypomastigotes were separated by four consecutive centrifugation rounds as before. The cell-free conditioned medium was centrifuged in a Beckman Optima XL-100K ultracentrifuge using a Type 70 Ti rotor at 31,000 rpm 98,914 xg (max) for 90min. Pellet was resuspended in 500μl of TBS and the supernatants were precipitated with cold acetone overnight at 4°C and resuspended in 500μl of TBS. All aliquots were cracked and developed by Western blots.

### Transmission Electron Microscopy (TEM)

Microvesicles harvested either by Exoquick (FBS-free) or ultracentrifugation were fixed with 4% EM-grade PFA in TEM-buffer (50mM HEPES, pH6.5) and 10μl of the suspension were absorbed in GO coated copper grids (200 mesh). Samples were pos-fixed in 1% EM-grade glutaraldehyde in TEM-buffer and rinsed with deionized water, then stained with 2% Uranyl acetate for 1min and dried at room temperature. Micrographs were acquired in a Carl Zeiss EM 109T transmission electron microscope operating at 80kV and equipped with a Gatan ES1000W (11 Mpixel) digital camera.

### PI-PLC treatment on trypomastigote microvesicles and conditioned medium

Trypomastigotes (200×10^6^) were rinsed and resuspended in 350μl of FBS-free RPMI-1640. Parasites were incubated for 2h at 37°C, 5% CO_2_ and then separated from the conditioned medium by consecutive centrifugation steps as above. The resulting conditioned medium (300μl) was split in half; one to serve as control and the other was supplemented with 0.5 enzymatic units of PI-PLC (Invitrogen) and incubated 30min at 37°C, 5% CO_2_. Then, both samples were supplemented with 10% FBS and microvesicles harvested using the Exoquick kit as above. Pellets and proteins precipitated from supernatant by cold acetone were cracked in a final volume of 100μl and cracked to a final volume of 100μl. Western blots were performed.

### PI-PLC treatment during trypomastigote shedding

Rinsed trypomastigotes (200×10^6^) were resuspended in 400μl of serum free RPMI-1640 and divided into four aliquots (each containing 50×10^6^ parasites in 200μl). Two aliquots served as controls. The other two were treated for 30 or 90min (PLC30 and PLC90) with 0.5 units of PI-PLC at 37°C in 5% CO_2_. At the indicated time, supernatant was depleted of cells and debris by centrifugation as above. Then, conditioned media were supplemented with 10% FBS and microvesicles were harvested using Exoquick as before. Pellets were cracked in a final volume of 100μl while supernatant proteins were precipitated with cold acetone and then cracked to a final volume of 100μl. Western blots were performed.

### Microvesicles harvest on extracellular medium from infected Vero cell cultures

Supernantants (5 ml) of Vero cell cultures in MEM supplemented with 4% FBS containing 10×10^6^ trypomastigotes/ml were taken at day 7-post-infection. Cells and debris were removed by centrifugation and microvesicles harvested from 500μl of the cell-free conditioned medium using the Exoquick kit as above. Pellets were cracked in a final volume of 100μl and supernatant proteins were precipitated with cold acetone and later cracked to a final volume of 100μl. Western blots were performed.

## Supporting Information

S1 Fig Methyl-β-cyclodextrin severely perturbs trypomastigote’s morphology. Trypomastigotes were incubated with methyl-β-cyclodextrin (MβCD) for 30min at different concentrations. (A) Phase contrast images for MβCD treatment at 0-30mM show how MβCD induces strong alterations to the trypomastigote structure. (B) Western blots for treatments with MβCD in concentrations between 0-5mM show that mucins are extracted from the trypomastigotes, in line with the microscopic observations. (C) Fluorescence microscopy of trypomastigotes treated with MβCD. Sialic acid is labeled in green and TS in red. Correlative with the increase in MβCD concentration, trypomastigotes disrupt their size, shape and dotted labeling of sialic acid (5mM) and TS (10mM). Even though MβCD disrupts lipid rafts, its effect on the trypomastigotes was not acceptable for continuing with further analysis. Bar: 5μm.
(TIF)

S2 Fig Sialic acid acceptors distribution in *T*. *cruzi* epimastigotes and metacyclic trypomastigotes. (A) Epimastigotes (upper panel) were sialylated by addition of recombinant TS and Neu5Az donors. Metacyclic trypomastigotes (lower panel) were incubated with Neu5Az donors. Parasites were analyzed by confocal microscopy. A dotted pattern can be observed in both parasite stages. (B) Epimastigotes sialylated as in (A) were assayed for DRM purification. Sialic acid was incorporated to diffuse bands from 30-60kDa corresponding to the expected size of the stage-specific mucins. Epimastigotes mucins were also contained in DRMs.
(TIF)

S3 Fig Mucins do not pass through a TS rich area in the membrane. A) Mucins from trypomastigotes were saturated with sialyl-lactose, exposed to Neu5Az donors for short periods (30sec to 15min) to label newly exposed sialyl acceptor sites and fixed immediately in 4% *p*-formaldehyde (PFA). Fluorescence images show that in none of the contexts did TS and mucins colocalized, indicating that mucins do not pass through a TS rich area for sialylation. Raw images are shown to avoid biased observations. B) PFA-Fixed trypomastigotes accept Neu5Az transferred by the TS shed from neighboring live trypomastigotes. Acceptor trypomastigotes were labeled with anti-TS 13G9 mAb and fixed. Then, fixed parasites were mixed 1:1 with live trypomastigotes in the presence of Neu5AzGal. At given times aliquots were fixed with PFA, processed with Phos-FLAG and revealed by immunofluorescence. Acceptor parasites were identified by TS staining (red fluorescence) and acquired sialyl residue by green fluorescence. Confocal images. Bar: 5μm.
(TIF)

S1 Table LC-MS-MS of DRM proteins. (XLSX)
