# Supplementary figures and images for: Sialic Acid Glycobiology Unveils Trypanosoma cruzi Trypomastigote Membrane Physiology
Source: PLoS Pathog. 2016 Apr 8;12(4):e1005559. doi: 10.1371/journal.ppat.1005559 (PMC4825991; doi:10.1371/journal.ppat.1005559)

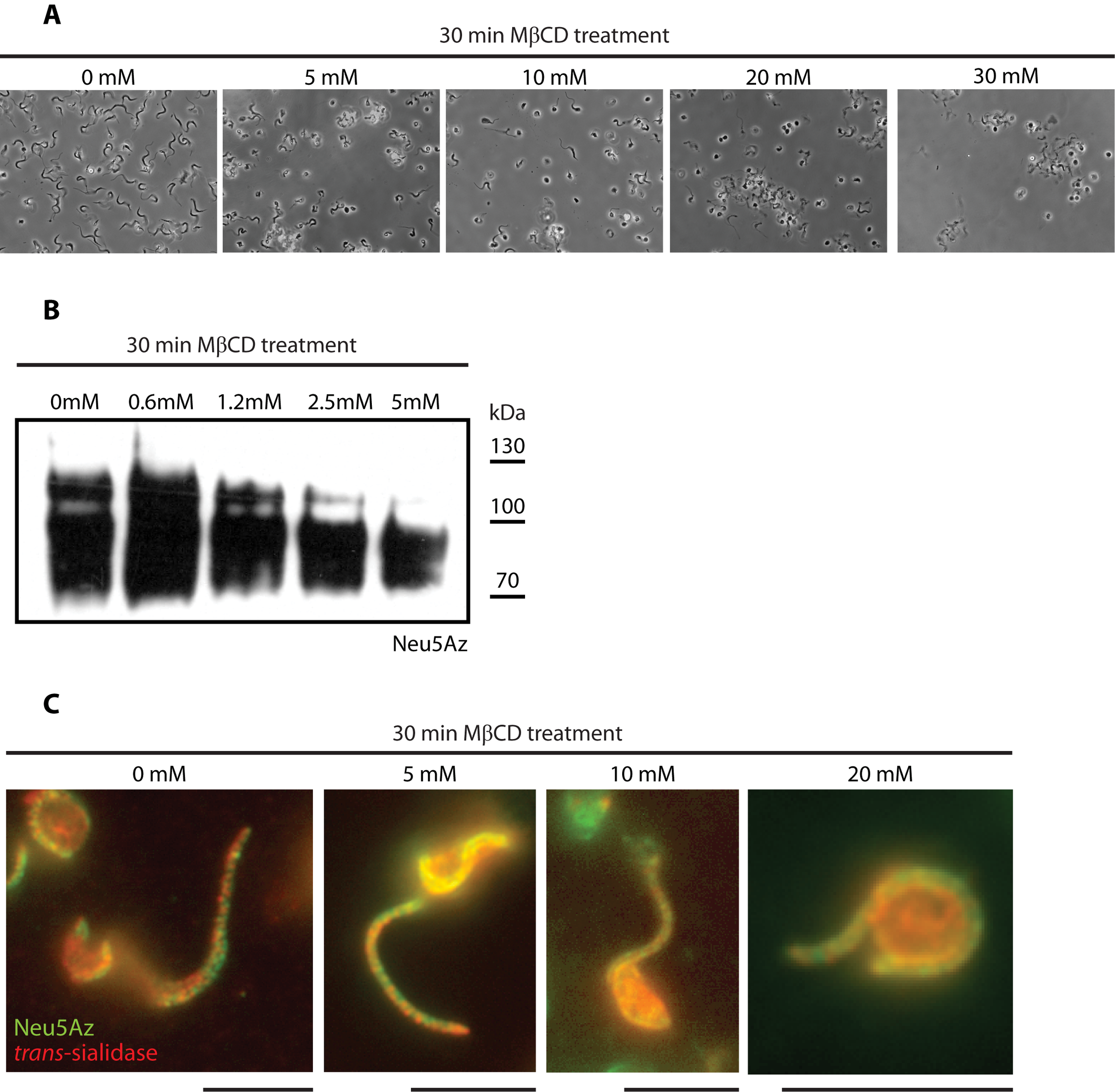

Supplement: S1 Fig — Trypomastigotes were incubated with methyl-β-cyclodextrin (MβCD) for 30min at different concentrations. (A) Phase contrast images for MβCD treatment at 0-30mM show how MβCD induces strong alterations to the trypomastigote structure. (B) Western blots for treatments with MβCD in concentrations between 0-5mM show that mucins are extracted from the trypomastigotes, in line with the microscopic observations. (C) Fluorescence microscopy of trypomastigotes treated with MβCD. Sialic acid is labeled in green and TS in red. Correlative with the increase in MβCD concentration, trypomastigotes disrupt their size, shape and dotted labeling of sialic acid (5mM) and TS (10mM). Even though MβCD disrupts lipid rafts, its effect on the trypomastigotes was not acceptable for continuing with further analysis. Bar: 5μm. (TIF) [file ppat.1005559.s001.tif]

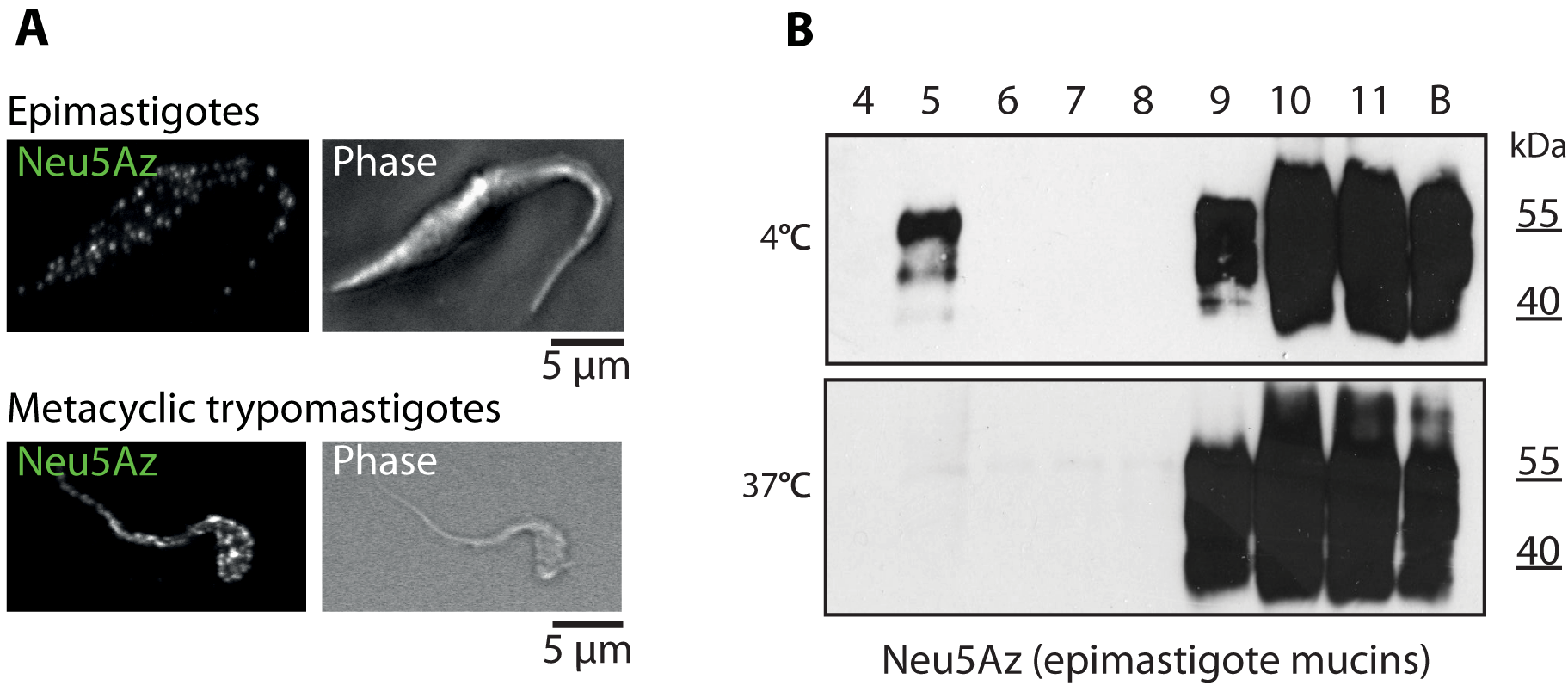

Supplement: S2 Fig — (A) Epimastigotes (upper panel) were sialylated by addition of recombinant TS and Neu5Az donors. Metacyclic trypomastigotes (lower panel) were incubated with Neu5Az donors. Parasites were analyzed by confocal microscopy. A dotted pattern can be observed in both parasite stages. (B) Epimastigotes sialylated as in (A) were assayed for DRM purification. Sialic acid was incorporated to diffuse bands from 30-60kDa corresponding to the expected size of the stage-specific mucins. Epimastigotes mucins were also contained in DRMs. (TIF) [file ppat.1005559.s002.tif]

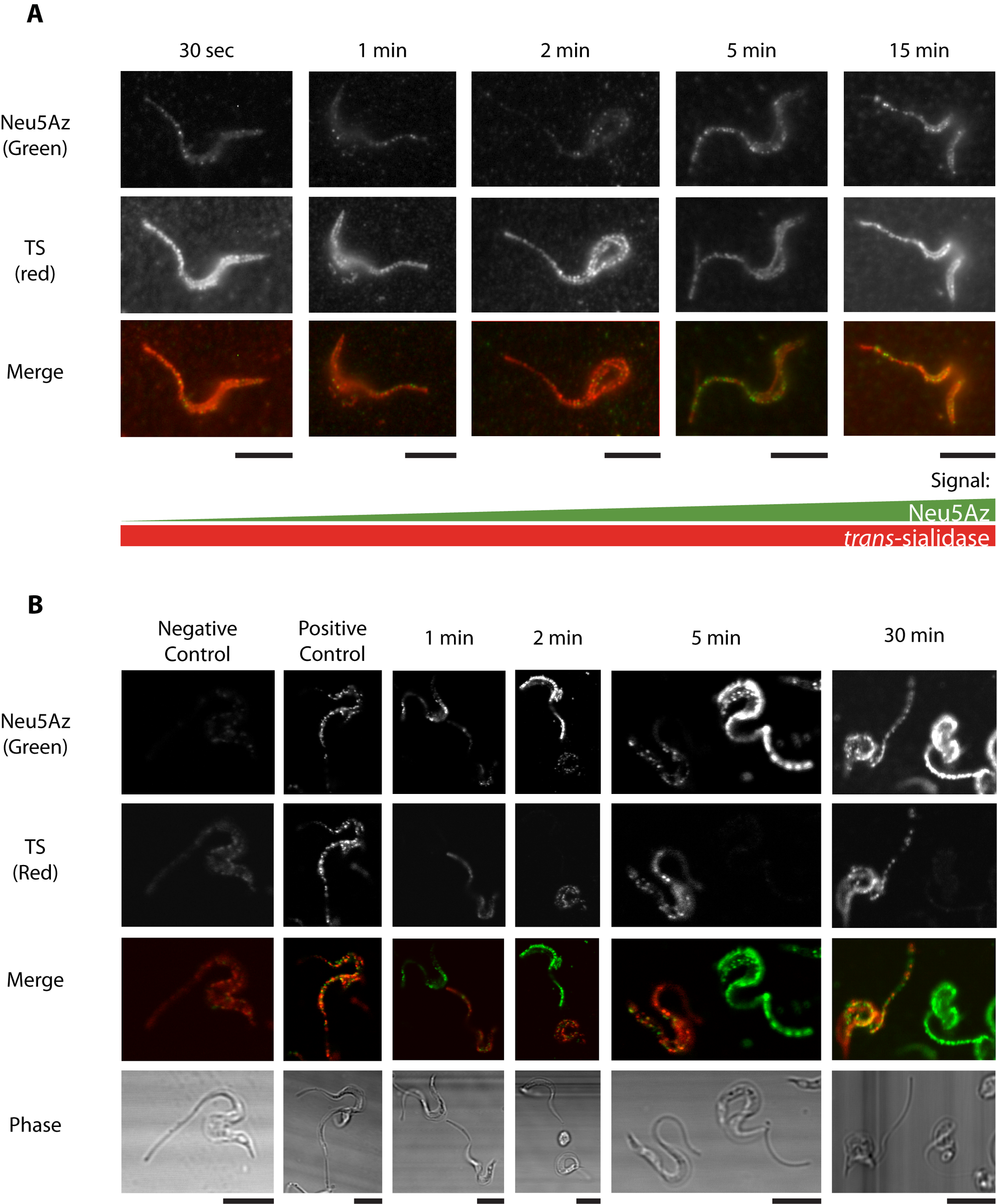

Supplement: S3 Fig — A) Mucins from trypomastigotes were saturated with sialyl-lactose, exposed to Neu5Az donors for short periods (30sec to 15min) to label newly exposed sialyl acceptor sites and fixed immediately in 4% p-formaldehyde (PFA). Fluorescence images show that in none of the contexts did TS and mucins colocalized, indicating that mucins do not pass through a TS rich area for sialylation. Raw images are shown to avoid biased observations. B) PFA-Fixed trypomastigotes accept Neu5Az transferred by the TS shed from neighboring live trypomastigotes. Acceptor trypomastigotes were labeled with anti-TS 13G9 mAb and fixed. Then, fixed parasites were mixed 1:1 with live trypomastigotes in the presence of Neu5AzGal. At given times aliquots were fixed with PFA, processed with Phos-FLAG and revealed by immunofluorescence. Acceptor parasites were identified by TS staining (red fluorescence) and acquired sialyl residue by green fluorescence. Confocal images. Bar: 5μm. (TIF) [file ppat.1005559.s003.tif]
